# Lymphocyte activation gene 3 (LAG3) protein expression on tumor-infiltrating lymphocytes in aggressive and *TP53*-mutated salivary gland carcinomas

**DOI:** 10.1007/s00262-020-02551-6

**Published:** 2020-03-30

**Authors:** Christoph Arolt, Moritz Meyer, Vanessa Ruesseler, Lisa Nachtsheim, Nora Wuerdemann, Thomas Dreyer, Stefan Gattenlöhner, Claus Wittekindt, Reinhard Buettner, Alexander Quaas, Jens Peter Klussmann

**Affiliations:** 1 Institute of Pathology, Medical Faculty, University of Cologne, Kerpener Str. 62, 50931 Cologne, Germany; 2 Department of Otorhinolaryngology, Head and Neck Surgery, Medical Faculty, University of Cologne, Cologne, Germany; 3 Jean-Uhrmacher-Institute for Otorhinolaryngological Research, University of Cologne, Cologne, Germany; 4 Institute of Pathology, Medical Faculty, University of Giessen and Marburg, Giessen, Germany; 5 Department of Otorhinolaryngology, Head and Neck Surgery, Medical Faculty, University of Giessen and Marburg, Giessen, Germany

**Keywords:** Salivary gland carcinoma, LAG3, *TP53*, Tumor micro-environment, Prognosis

## Abstract

Salivary gland carcinomas (SGCs) are rare and can be subdivided into distinct entities, some of which confer a poor prognosis. As targets for effective systemic therapy are warranted, some studies investigated the role of immune-checkpoint proteins PD-L1 and CTLA-4 in SGC. Our study depicts the expression of lymphocyte activation gene 3 **(**LAG3) in a test cohort and a larger validation cohort, totaling 139 SGCs. LAG3 is expressed on tumor-infiltrating lymphocytes (TILs), mediates T cell exhaustion and is subject to numerous currently recruiting clinical studies. Overall, one-third of SGCs were infiltrated by LAG3-expressing TILs with a strikingly high concordance between the test cohort and the validation cohort (30% and 28.2%, respectively). In the validation cohort, entity-wise LAG3 expression frequencies were highly variable. The highest rates were observed in salivary duct carcinoma (SDC; 66.7%) and adenocarcinoma not otherwise specified (ANOS; 50.0%). We observed LAG3 expression on effector T cells and in smaller frequencies also on FOXP3− T helper cells and FOXP3+ Tregs. LAG3 expression significantly correlated with advanced nodal metastases, cytotoxic T cell infiltrate and *TP53* mutations. In the group of adenoid cystic carcinomas, LAG3 expression was also associated with a shorter event-free survival (EFS). Tumors with *TP53* nonsense mutations (*TP53* null type) exhibited higher LAG3 frequencies and a shorter EFS compared to *TP53* wild type. This is the first report of LAG3 expression in SGC, a promising target for immunotherapy. LAG3 blockage could be distinctly applicable for SDC and ANOS, two SGC types with a particularly poor outcome.

**Electronic supplementary material:**

The online version of this article (10.1007/s00262-020-02551-6) contains supplementary material, which is available to authorized users.

## Introduction

Setting boundaries to T cell activity through inhibitory co-receptors (IR) is part of the physiological regulation of immune responses. Conversely, in the setting of a sustained and uncontrolled local presentation of neoantigens by tumor cells (TC), the upregulation of IRs can lead to T cell exhaustion in the tumor micro-environment (TME) [1]. Blockage of these inhibitory stimuli is the principle of checkpoint inhibitors. Most prominently among this group, inhibitors aiming at programmed cell death protein 1 and its ligand 1 (PD-1/PD-L1) have recently led to a breakthrough in targeted cancer therapy and are nowadays used for the treatment for a multitude of neoplastic diseases, including nonsmall cell lung cancer (NSCLC), melanoma and urothelial carcinoma [2]. Despite the success of a PD-1/PD-L1 blockage in some patients, the majority of patients do not respond [3]. This led to the therapeutic exploration of alternative IRs, including lymphocyte activation protein 3 (LAG3).

LAG3, a co-receptor of the T cell receptor, is structurally highly homologous to CD4 and shares its main ligand MHC class II, binding to it with a much higher affinity than CD4 [4]. In melanomas and colorectal cancer, LAG3 is predominantly expressed on FOXP3+ regulatory T cells that exert an immunosuppressive function through release of TGF-ß and IL10 in the TME [5]. A co-expression of LAG3 and PD-L1 on tumor-infiltrating lymphocytes (TILs) has been observed in several tumor types [6]. Dual blockage of LAG3 and PD-L1 with monoclonal antibodies led to increased CD4- and CD8-positive TILs and tumor clearance compared to monotherapy in mice models [7]. Head and neck squamous cell carcinoma (HNSCC) and colorectal carcinoma with LAG3-expressing TILs were associated with lymph node metastasis, larger tumor size and shorter overall survival in the case of nodal status-negative HNSCC [8].

At present, numerous clinical trials investigate the efficacy of LAG3 blockage, enrolling patients with a broad spectrum of neoplastic diseases (e.g., head and neck carcinomas, melanoma, colorectal carcinoma, NSCLC: NCT01968109).

The relatively rare carcinomas of the salivary glands (SGC) make up fewer than 0.5% of all cancers and can be subdivided into distinct histologic types, with mucoepidermoid carcinoma (MEC) as the most frequent subgroup. Entity-specific genomic translocations with an oncogenic driver potential were revealed for adenoid cystic carcinomas (AdCys), MEC and more recently, secretory carcinomas and acinic cell carcinomas [9]. Currently, with the exception of secretory carcinomas, these genomic alterations cannot yet be therapeutically exploited [10]. The biological aggressiveness is overall moderate compared to other carcinomas, but it varies significantly across different subtypes. For instance, the prognosis of salivary duct carcinomas (SDC) is poor (mean overall survival: 56 months) [11]. Therapy consists of complete excision, followed by stage- and risk-adapted radiotherapy. In a setting of irresectability, nodal or distant metastasis, prognosis is unfavorable (67% and 34% 5-year OS, for regional and distant metastasis, respectively) and only untargeted chemotherapy with short response duration is at hand for most entities as first-line treatment. The demand for therapeutic targets prompted the exploration of inhibitory immune-checkpoint molecules expression in SGC: Recent studies presented divergent results for PD-L1 expression on tumor cells and TILs [12–16]. PD-L1 positivity was associated with poor outcome and higher UICC stage (Union International Contre le Cancer) [12, 17]. Also, programmed death ligand 2 (PD-L2) and cytotoxic T-lymphocyte-associated antigen 4 (CTLA-4) were expressed on SGC tumor cells at frequencies of 63% and 74.1%, respectively, whereby PD-L2 expression was associated with a shorter progression-free survival [13]. The first clinical study with immune-checkpoint inhibitors in SGC applied the PD-1 inhibitor pembrolizumab in a metastatic/recurrent disease setting. A relatively high rate of responders (12%) and a manageable safety profile raised hopes for immunotherapeutic options in SGC [18]. Another comparable phase II study reported a slightly higher response rate of 16% for SGC (only partial responses) [19]. Also recently, a phase II trial demonstrated encouraging effectiveness of the PD-1 inhibitor nivolumab in combination with CTLA-4 inhibitor ipilimumab in SGC [20]. Another study which evaluates the therapeutic use of nivolumab alone is ongoing (NCT03132038). While a series of studies investigated the role of PD-L1, PD-L2 and CTLA-4, little is known about the presence of other IRs in SGC.

Somatic *TP53* mutations are considered to be the most frequent genetic alterations in cancer cells of different origin. TP53 immunohistochemistry (IHC) is able to detect and discriminate between nonsense (indel, stopgain and alternative splicing) and missense (nonsynonymous) mutations: While the former lead to a complete loss of TP53 protein expression, the latter usually result in nuclear TP53 protein overexpression [21]. Missense mutations occur more often (87.9%) than nonsense or silent mutations, but either can lead to a loss of function. Intact *TP53* acts as a tumor suppressor gene, initiating cell cycle arrest and apoptosis under genotoxic stress. A deregulated proliferational capacity due to a loss of TP53 function seems to be the main selective advantage for *TP53* mutations [22] and allows for a higher mutational burden [23]. Consecutively, a higher number of neoantigens presented on the cell surface induce an immune response. A study investigating the role of *TP53* in human NSCLC cell lines also revealed that certain *TP53* mutations lead to a direct, micro-RNA-mediated upregulation of PD-L1, suggesting a mechanism to dampen proinflammatory mutational effects [24]. In SGC, *TP53* mutations occur at an overall frequency of 30% [25], with higher frequency in more aggressive carcinomas. Additionally, frequency of *TP53* mutations correlated with the tumor mutational burden, which indicates a higher immunogenic potential [26]. In SGC as in several other carcinomas, *TP53* mutations have been associated with a significantly shorter survival [27].

For the first time, the present study depicts the expression of LAG3 in a large cohort of SGC considering different well-defined subtypes in order to forge new paths for effective systemic therapy.

## Materials and methods

### Cohort and TMA preparation

For the test cohort, 33 pre-therapeutic tissue samples from primary salivary gland carcinomas were collected from the archives of the Institute of Pathology, University Hospital of Giessen. A validation cohort was then set up from 106 samples of the same kind from the archives of the Institute of Pathology, University Hospital of Cologne. All patients were treated at the Department of Oto-Rhino-Laryngology, Head and Neck Surgery of the University of Giessen between 2011 and 2017 or at the Department of Oto-Rhino-Laryngology, Head and Neck Surgery of the University of Cologne between 1998 and 2018.

Four tissue cylinders per case with a diameter of 1.2 mm were punched out from one tumor-bearing formalin-fixed and paraffin-embedded (FFPE) block using a self-constructed semiautomated precision instrument. The cylinders were then transferred to an empty paraffin block. The tissue micro-arrays (TMAs) each consisted of 96 cylinders representing 24 cases. Fluorescence in situ hybridization (FISH) and IHC were performed on freshly cut 4-μm TMA sections.

### Fluorescence in situ hybridization

We retrospectively reviewed initial diagnosis of the validation cohort using FISH to detect entity-specific translocations of MYB, MAML2 and ETV6 genes (ZytoLight SPEC MYB, ZytoLight SPEC MAML2, ZytoLight SPEC ETV6) for detection of AdCy, MEC and secretory carcinomas, respectively, all dual-color break apart probes; 4-μm tissue sections were transferred to adhesive slides and heated to 60 °C for paraffin removal, followed by semiautomated deparaffinization and protein digestion (VP2000 processor system, Abbott Molecular, Wiesbaden, Germany; Vysis IntelliFISH Universal FFPE Tissue Pretreatment Protease; Abbott Molecular, Wiesbaden, Germany). Next, tissue sections were denatured at 75 °C for 10 min and hybridized with the probe at 37 °C overnight, followed by DAPI staining. Surrounding nontumor cells were used as negative control.

### Immunohistochemistry

TMA sections of the test cohort and the validation cohort were stained with the anti-LAG3 rabbit IgG monoclonal antibody D2G40 (Cell Signaling Technology, the Netherlands; dilution 1:300). TMA sections of the validation cohort were further stained with mouse monoclonal antibody C8/144B (Dako/Agilent, USA; dilution 1:200) and mouse monoclonal antibody DO-7 (Dako/Agilent, USA; dilution 1:1200) for the expression of CD8 and TP53, respectively. All IHC stainings were performed using a Leica BOND-MAX stainer (Leica Biosystems, Germany) in accordance with the manufacturer’s protocol.

Triple stainings were performed on a Ventana Discovery Ultra automated staining system. The following primary antibodies were used: rabbit anti-LAG3 IgG monoclonal antibody D2G40 (Cell Signaling Technology, the Netherlands; dilution 1:300), mouse anti-CD8 monoclonal antibody C8/144B (Dako/Agilent, USA; dilution 1:200), mouse anti-CD68 monoclonal antibody PG-M1 (Dako/Agilent, USA; dilution 1:400), mouse anti-FOXP3 monoclonal antibody 236A/E7 (Abcam, UK; dilution 1:100), rabbit anti-CD4 monoclonal antibody 4B12 (Roche, Switzerland, ready to use). After conjugation with an antibody-bound enzyme (horseradish peroxidase or alkaline phosphatase), detection was carried out using DISCOVERY Silver kit (LAG3), DISCOVERY Yellow kit (FOXP3, CD8), DISCOVERY Teal kit (CD68), DISCOVERY Red Kit (CD4, all Ventana/Roche, Switzerland)). Counterstaining was done with hematoxylin and bluing reagent.

Monocolor IHC stainings for LAG3 and CD8 were assessed semiquantitatively in a three-level scheme as follows: LAG 3: negative (< 1% TILs stained), low (1–5% TILs stained) and high (> 5% TILs stained); CD8: negative (< 10 TILs per core), low (10–100 TILs per core), high (> 100 TILs per core). In the case of heterogeneous staining within one case, the highest observed category was assigned. For reasons of clarity and binary analysis, cases with “LAG3 low” and “LAG3 high” were summarized as “LAG3 expressed” versus “not expressed” and cases declaring “CD8 high” were defined as “inflamed” in contrast to “CD8 low” and “CD8 negative,” which were termed “not inflamed.” The cutoff at 1% has previously been used to assess LAG3 expression in a clinical trial with using LAG3 blockage for the treatment for malignant melanoma [28]. In the present study, we used an IHC-based evaluation of *TP53* mutations: A mosaic-like staining pattern corresponds to a *TP53* wild type (WT). Mutational expression patterns were termed “null type” (NT) for absent nuclear staining and “overexpressed” (OT) for a nuclear staining of > 90% of tumor cells with higher intensity than stromal cells. These two patterns have previously been associated with nonsense and missense mutations, respectively [21, 29].

### Statistical analysis

Statistical analysis of the data from the validation cohort was performed using SPSS statistical software (IBM SPSS 25.0, Armonk, NY). Survival curves were generated with RStudio (R Foundation for Statistical Computing, Vienna, Austria.) and the R package survminer [30]. Interdependence between the assessed IHC markers and clinical characteristics was evaluated using Fisher’s exact test or Pearson’s Chi-square test where appropriate. Event-free survival (EFS) was defined as the period of time from initial diagnosis to clinically documented relapse or death. In case of a follow-up shorter than 10 years, survival time was censored. Additional right censoring was done after 10 years of event-free follow-up. Survival probability was estimated with the Kaplan–Meier model, and differences between variable levels were tested for significance using the log-rank test. Cox regression analysis was used to estimate the hazard ratios (HR) for uni- and multivariate analysis. The latter included age category (< or ≥ 65 years), nodal status (positive vs. negative), T-stage, therapy, consumption of alcohol and nicotine as covariates in addition to the target variable. Other target variables were excluded from multivariate analysis due to significant interdependencies. Statistical tests were considered significant if the two-sided alpha error was estimated below 0.05 and the calculated value remained within a 95% confidence interval (CI).

## Results

**Fig. 1:**
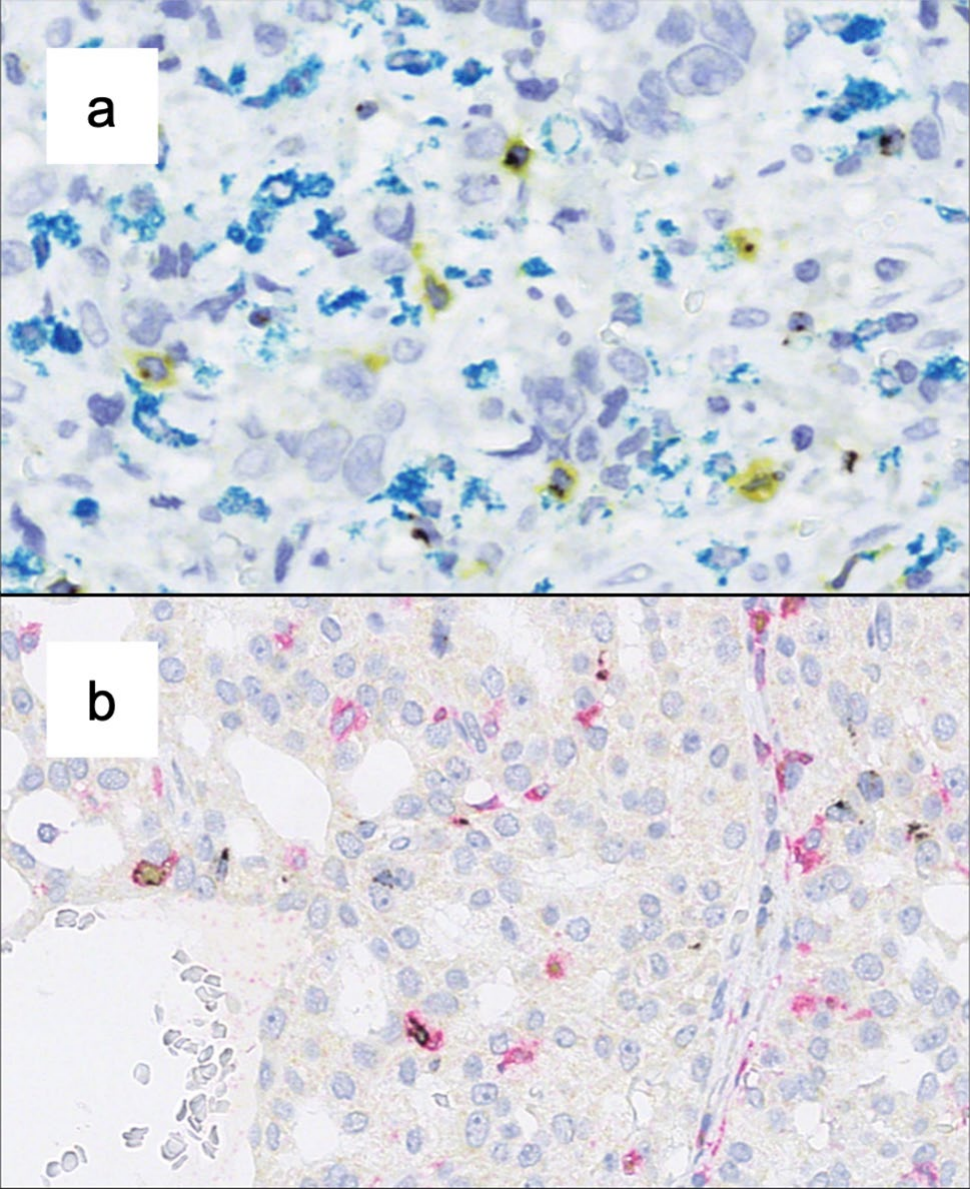
Exemplary triple-color IHC demonstrating co-expression of immunological markers with LAG3 (black). **a** Co-expression with CD8 (yellow) but not with CD68 (teal). **b** Co-expression of LAG3 (black) with CD4 (red) or with CD4 and FOXP3 (red and yellow)

**Fig. 2:**
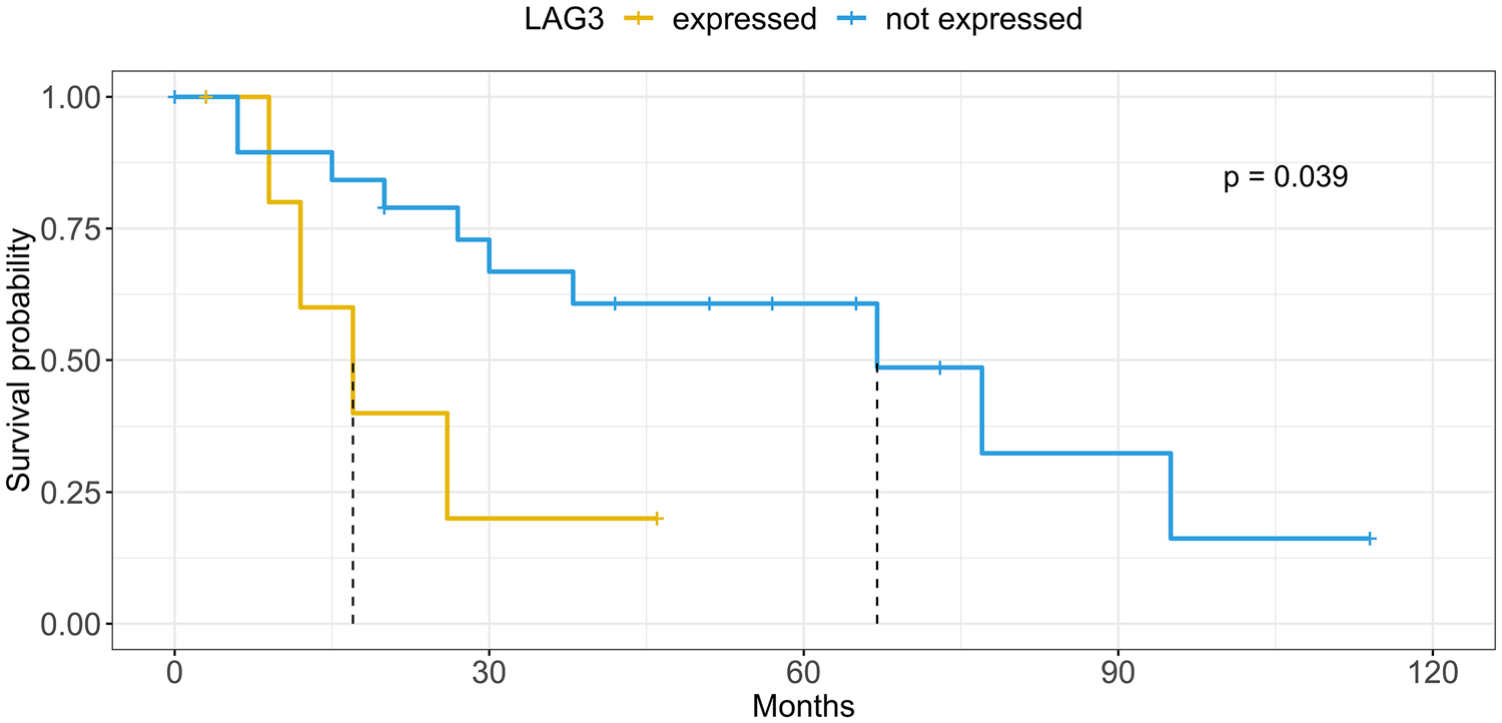
10-year EFS of the AdCy group stratified by LAG3 expression status. Dashed lines indicate median EFS for each stratum

**Fig. 3:**
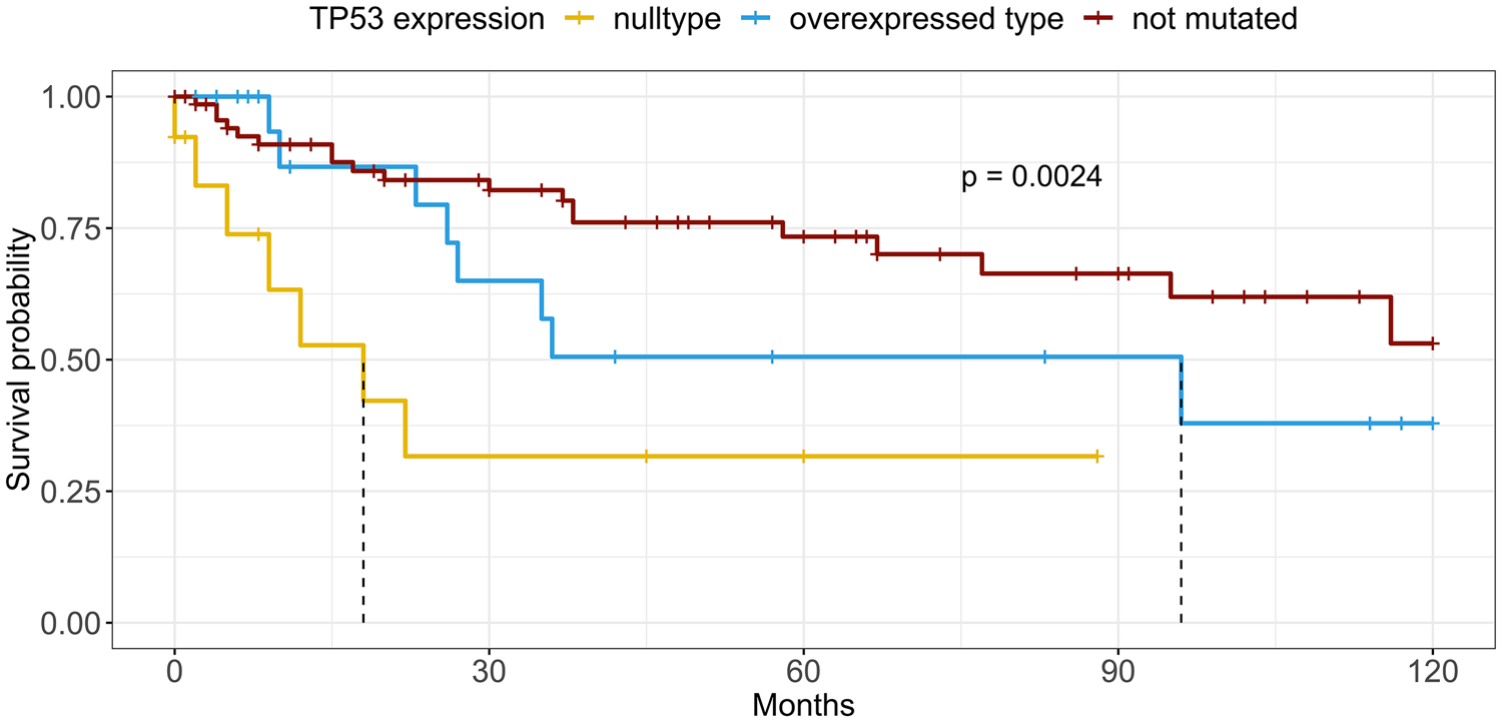
10-year EFS of the overall cohort stratified by *TP53* mutation type (blue and yellow lines) and nonmutated (wild type) tumors (red line). Dashed lines indicate median EFS for each stratum

LAG3, CD8 and TP53 expressions per carcinoma type are displayed in Table 1. The relationship between the above-mentioned markers and clinical characteristics for the validation cohort are presented in Table 2. Exemplary multicolor IHC stainings are shown in Fig. 1. Survival data can be taken from Table 3, while the estimates of the 10-year EFS by the Kaplan–Meier model are shown in Figs. 2 and 3. Interdependencies for LAG3, CD8 and *TP53* are demonstrated in supplementary Table 1.

**Table 1:** Overview of LAG3 positivity, significant CD8+ inflammation, TP53 mutation and main clinicopathological parameters for each carcinoma entity; displayed as percentage of entity total

	Adenoid cystic	Mucoepidermoid	Acinic cell	Adeno NOS	Salivary duct	Epithelial-Myoepithelial	Secretory	Total
Test cohort								
Count	5	8	6	8	6	0	0	33
LAG3 positivity	0%	37.5%	50%	12.5%	50%	0.0%	0.0%	30.3%
Validation cohort								
Count	29	27	9	19	8	6	7	106
LAG3 positivity	20.7%	18.5%	22.2%	50.0%	66.7%	0.0%	20.0%	28.2%
CD8+ inflammation	13.8%	25.9%	33.3%	47.4%	33.3%	0.0%	14.3%	25.5%
TP53 mutation	22.2%	24.0%	25.0%	47.4%	66.7%	16.7%	14.3%	30.7%
Age								
< 65 years	72.4%	80.8%	88.9%	57.9%	11.1%	33.3%	71.4%	65.7%
≥ 65 years	27.6%	19.2%	11.1%	42.1%	88.9%	66.7%	28.6%	34.3%
Gender								
m	34.5%	25.9%	55.6%	63.2%	66.7%	66.7%	57.1%	45.3%
w	65.5%	74.1%	44.4%	36.8%	33.3%	33.3%	42.9%	54.7%
T								
T1	21.4%	25.0%	44.4%	5.6%	11.1%	33.3%	57.1%	23.8%
T2	25.0%	33.3%	11.1%	16.7%	22.2%	33.3%	28.6%	24.8%
T3	14.3%	20.8%	22.2%	33.3%	22.2%	16.7%	14.30%	20.8%
T4a	25.0%	8.3%	22.2%	38.9%	33.3%	16.7%	0.0%	21.8%
T4b	14.3%	12.5%	0.0%	5.6%	11.1%	0.0%	0.0%	8.9%
N								
N0	66.7%	76.0%	62.5%	27.8%	25.0%	100.0%	85.7%	61.6%
N1	14.8%	0.0%	12.5%	11.1%	12.5%	0.0%	14.3%	9.1%
N2a	3.7%	0.0%	0.0%	5.6%	0.0%	0.0%	0.0%	2.0%
N3	14.8%	24.0%	25.0%	55.6%	62.5%	0.0%	0.0%	27.3%
UICC								
I	18.5%	25.0%	25.0%	5.9%	12.5%	33.3%	42.9%	20.6%
II	25.9%	25.0%	12.5%	5.9%	12.5%	33.3%	28.6%	20.6%
III	14.8%	20.8%	37.5%	11.8%	0.0%	16.7%	28.6%	17.5%
IVa	11.1%	4.2%	0.0%	17.6%	12.5%	16.7%	0.0%	9.3%
IVb	25.9%	20.8%	25.0%	47.1%	62.5%	0.0%	0.0%	27.8%
IVc	3.7%	4.2%	0.0%	11.8%	0.0%	0.0%	0.0%	4.1%

**Table 2:** Overall distribution of LAG3, CD8 and *TP53* mutations depending on clinical/tumor criteria; percentages per variable are displayed in parentheses

	LAG3			CD8+ inflammation			TP53		
	Negative	Positive	*p*	Not inflamed	Inflamed	*p*	Not mutated	Mutated	*p*
Age group									
< 65 years	52 (71.2)	15 (51.7)		52 (66.7)	17 (63)		49 (71)	16 (51.6)	
≥ 65 years	21 (28.8)	14 (48.3)		26 (33.3)	10 (37)		20 (29)	15 (48.4)	
Gender									
m	35 (47.3)	12 (41.4)		35 (44.3)	13 (48.1)		30 (42.9)	16 (51.6)	
w	39 (52.7)	17 (58.6)		44 (55.7)	14 (51.9)		40 (57.1)	15 (48.4)	
Alcohol									
No	49 (80.3)	20 (83.3)		55 (83.3)	16 (76.2)		49 (84.5)	18 (72)	
Yes	10 (16.4)	4 (16.7)		10 (15.2)	4 (19)		9 (15.5)	5 (20)	
Abuse	2 (3.3)	0 (0)		1 (1.5)	1 (4.8)		0	2 (8)	
Nicotine									
No	50 (80.6)	18 (78.3)		55 (83.3)	15 (71.4)		48 (82.8)	18 (72)	
Yes	12 (19.4)	5 (21.7)		11 (16.7)	6 (28.6)		10 (17.2)	7 (28)	
Therapy									
Surgery	31 (46.3)	8 (30.8)		34 (47.2)	7 (30.4)		33 (52.4)	6 (21.4)	
+RTX/CTX	36 (53.7)	18 (69.2)		38 (52.8)	16 (69.6)		30 (47.6)	22 (78.6)	
T									
T1	21 (30)	2 (7.1)		19 (25.7)	5 (18.5)		23 (33.8)	0	**0.02**
T2	14 (20)	11 (39.3)		19 (25.7)	6 (22.2)		15 (22.1)	8 (28.6)	
T3	15 (21.4)	4 (14.3)		16 (21.6)	5 (18.5)		13 (19.1)	7 (25)	
T4a	14 (20)	8 (28.6)		12 (16.2)	10 (37)		12 (17.6)	9 (32.1)	
T4b	6 (8.6)	3 (10.7)		8 (10.8)	1 (3.7)		5 (7.4)	4 (14.3)	
N									
N0	47 (69.1)	11 (39.3)	**0.001**	50 (67.6)	11 (44)	**0.008**	48 (71.6)	10 (35.7)	**<0.001**
N1	8 (11.8)	1 (3.6)		8 (10.8)	1 (4)		7 (10.4)	2 (7.1)	
N2a	0	2 (7.1)		0	2 (8)		2 (3)	0	
N3	13 (19.1)	14 (50)		16 (21.6)	11 (44)		10 (14.9)	16 (57.1)	
UICC stadium									
I	17 (25.4)	2 (7.4)	**0.009**	16 (22.2)	4 (16)		19 (28.4)	0	**<0.001**
II	13 (19.4)	7 (25.9)		15 (20.8)	5 (20)		14 (20.9)	5 (19.2)	
III	14 (20.9)	1 (3.7)		16 (22.2)	1 (4)		13 (19.4)	3 (11.5)	
IVa	7 (10.4)	2 (7.4)		6 (8.3)	3 (12)		8 (11.9)	1 (3.8)	
IVb	15 (22.4)	12 (44.4)		18 (25)	9 (36)		10 (14.9)	16 (61.5)	
IVc	1 (1.5)	3 (11.1)		1 (1.4)	3 (12)		3 (4.5)	1 (3.8)	

RTX: radiotherapy, CTX: chemotherapy

**Table 3:** Overall and entity-specific survival analysis

	*N*	Kaplan–Meier model		Univariate Cox regression		Multivariate Cox regression
		Median EFS	*p*	HR	*p*	
Overall						
LAG3 score						
<1% TILs	69					
1–4% TILs	14	116	0.350	1.64	0.327	
≥ 5% TILs	14	38	0.400	1.43	0.477	
LAG3 status						
Negative	69					
Positive	28	116	0.275	1.53	0.279	
TP53 type						
Wild type	64	116				
Null type	12	18	**0.014**	3.20	**0.015**	*p* = 0.065
Overexpression	18	96	0.962	1.02	0.967	
Adenoid cystic carcinoma						
LAG3 score						
< 1% TILs	20	67				
1–4% TILs	4	12	**0.004**	6.85	**0.011**	
≥ 5% TILs	2	17	0.702	1.50	0.704	
LAG3 status						
Negative	20	67				
Positive	6	17	**0.039**	3.46	0.053	*p* = 0.091
TP53 type						
Wild type	18	67				
Null type	2	9	**0.001**	25.76	**0.010**	
Overexpression	4	27	0.818	1.20	0.824	

Significant *p* values are printed in bold type. For multivariate regression, only *p* value below 0.1 are displayed

### Distribution of LAG3, CD8 and TP53 expressions

In the test cohort, ten out of 33 SGC displayed an infiltration by LAG3-expressing TILs. While 50% of the acinic cell carcinomas and SDC were LAG3 positive, AdCy and ANOS showed no LAG3 staining.

In total, 29 of 106 SGCs of the validation cohort were infiltrated by LAG3-expressing TILs, which confirms the frequency of LAG3 expression obtained from the test cohort (30.3% and 28.2% for test and validation cohort). The frequency of LAG3 positivity ranged from 0% for epithelial–myoepithelial carcinomas to 66.7% for SDC. Overall, UICC stadium, frequency and extent of lymph node metastasis were higher in the LAG3-positive group (*p* = 0.009 and 0.001, respectively).

LAG3 positivity was associated with an inflamed TME (< 0.001). This concordance was also observed in each single histologic subgroup. Overall, *TP53* mutations occurred in 31 of 101 of the cases (30%), with frequencies ranging from 66.7% for SDC to 14.3% for secretory carcinomas. LAG3 positivity of TILs was more often seen in *TP53* mutated than in not mutated carcinomas (*p* < 0.001), and it varied across mutation subtypes: A LAG3-positive immune infiltrate was detected in 18%, 42.1% and 66.7% for WT, OT and NT, respectively. In our cohort, *TP53*-mutated tumors had been assigned to a higher T and N category (*p* values: = 0.02 and < 0.001) as well as to a higher UICC-stage (*p* < 0.001).

Compared to other entities, AdCy cases (*n* = 29) were less frequently LAG3 positive (20.7%; mean: 28.2%), inflamed (13.8%; mean 25.5%) and *TP53* mutated (22.2%; mean: 30.7%). No significant associations between LAG3 expression and other clinical parameters were observed.

### Immunophenotyping of LAG3-expressing immune cells

Studies regarding different types of carcinomas have reported LAG3 expression in distinct T cell subsets. Hence, using state-of-the-art multicolor IHC on two exemplary TMA slides, we evaluated co-staining of LAG3 with T-cell-related antigens CD4, CD8 and FOXP3 as well as with CD68 for detection of macrophages.

LAG3 was mainly expressed on CD8+ positive and, in a smaller frequency, also on CD4+ TILs.

Occasionally, also co-expression of LAG3, CD4 and FOXP3 was observed. LAG3 expression on macrophages was negligible.

### Survival analysis

With a median follow-up of 39 months, locally advanced (T3, T4) tumors and tumors with lymphatic metastases exhibited a decreased EFS (*p* = 0.004 and *p* = 0.032, respectively). With all carcinoma types pooled, neither the expression of LAG3 nor of CD8 on TILs had a significant prognostic impact. In contrast, AdCy cases with LAG3-positive TILs in the TME exhibited a shorter median EFS (17 vs. 67 months, *p* = 0.039) and a trend toward an elevated HR in the univariate and multivariate analysis (univariate: HR 3.46, *p* = 0.053, CI 0.98–12.16; multivariate: HR 5.03, *p* = 0.091, CI 0.77–32.83). *TP53*-mutated carcinomas with *TP53* NT were associated with a shorter median EFS (18 months) compared to the *TP53* WT tumors (116 months, *p* = 0.014), whereas the *TP53* OT showed no statistical tendency toward poorer EFS (96 months, *p* = 0.962, Fig. 1). Moreover, NT-mutated cases had an elevated HR of 3.198 in the univariate analysis (*p* = 0.015, CI 1.26–8.16) and a trend toward a higher HR in multivariate analysis (HR 3.94, *p* = 0.068, CI 0.92–16.90). None of the factors reached significance in the multivariate cox regression.

In AdCy, *TP53* NT mutation was associated with an elevated HR in univariate analysis (HR 25.76, *p* = 0.01, CI 2.20–301.11) and decreased median EFS (9 vs. 67 months, *p* = 0.001, Fig. 2). The multivariate analysis as well as the comparison between *TP53*-mutated and not-mutated cases did not present significant differences.

## Discussion

Therapeutic options for metastatic and recurrent SGC are still sparse, even though several studies have shed light into their mutational landscape: Few recurrent actionable genomic alterations have been reported in certain subtypes, but most patients cannot be offered a targeted therapy nowadays. While other tumors have been extensively profiled for the expression of immunosuppressive molecules in the past years, only a few studies have depicted the TME of SGC. To our knowledge, we are the first to describe the importance of LAG3 in a bicentric approach with a test cohort and a large validation cohort of SGC with a significant number of cases across the different histotypes.

In our exploratory test cohort, 30.3% of all SGCs were infiltrated by LAG3-expressing TILs. Strikingly, this frequency was almost exactly confirmed by our validation cohort, which exhibited LAG3 expression on TILs in 28.2% of the cases. Even though the two cohorts revealed divergent frequencies for some tumor types, the prevalence of LAG3-expressing TILs in SDC, one of the most aggressive entities, was consistently high (50% and 66.7% for the test cohort and the validation cohort, respectively).

Our validation cohort can be separated in two prognostic groups: SDC and ANOS are often locally advanced tumors with lymph node metastasis and relatively poor prognosis. Tumors of other histologic groups which were included in the cohort tend to grow more indolently (e.g., MEC). While only approximately 20% of the latter were LAG3 positive, the former, aggressive tumor types were infiltrated by LAG3-positive TILs at high frequencies of 66.7% and 50%, respectively. This has direct translational relevance, as among others, patients with SGC are currently eligible for two clinical studies which evaluate the potential of LAG3 blockage either alone or in combination with nivolumab (NCT01968109, NCT02720068). For SDC and ANOS, new approaches for targeted therapies are warranted: While SDC is frequently *HER2* (*ERBB2*) amplified and recent clinical studies involving trastuzumab yielded promising results, response duration is limited [31]. In contrast, ANOS lacks elsewhere-prevalent oncogenic mutations, leaving cytotoxic regimen as sole therapeutic option for recurrent or metastatic cases [25].

But SGC types with more variable aggressiveness, for instance AdCy, can also recur, metastasize or be irresectable. For these tumor groups, the therapeutic blockage of LAG3 could be considered as well.

Previous studies have demonstrated that SGCs express PD-L1, PD-L2 and CTLA at varying frequencies of up to 86% [12, 13, 15, 16]. Also, checkpoint inhibitor therapy leads to dramatic responses in some patients [18, 20]. Together with the mentioned results, our findings suggest that inhibition of immune-checkpoint molecules could be an option for systemic therapy.

IRs are expressed in the complex interplay between tumor cells and the immune system. Many studies contributed to the concept that chronic, tumor cell-controlled inflammation results in a favorable micro-environment for tumor progression [32]. Among many other parameters, infiltration by CD8-positive T lymphocytes and *TP53* mutations was reported to play an important role in this context. Our data demonstrate that nonsense mutations (NT) of *TP53* lead to a higher frequency of LAG3 positivity compared to WT *TP53*. This loss of TP53 protein function results in higher immunogenicity due to an accumulation of somatic mutations. Furthermore, loss of *TP53* leads to an upregulation of another IR, PD-L1, through the lack of micro-RNAs miR-34a and miR-200 [24]. While cases with missense mutations (OT) were LAG3 positive in fewer cases than nonsense mutations (NT), their extent of LAG3-bearing lymphocytes was higher (36.8%, 16.7% and 9% for OT, NT and WT, respectively). Missense mutations have been demonstrated to possess gain-of-function (GOF) properties, possibly resulting in very different forms of immunomodulation [33, 34]. Additionally, as shown in HNSCC, neoantigens derived from mutated *TP53* itself are detected by cytotoxic and helper T cells [35]. We hypothesize that the different immunomodulatory potential of missense mutations might lead to a more variable LAG3 expression than nonsense mutations. Whether certain GOF mutations or loss of TP53 could have direct, distinct impacts on LAG3 regulation should be further investigated.

An increasing CD8 expression has widely been correlated with antitumor immune response and tumor regression. Further, expressions of IRs and CD8 correlate in a variety of tumors, suggesting a means to dampen antitumor inflammatory responses [36]. In the present study, CD8-positive immune infiltration strongly correlated with LAG3 expression (*p* < 0.001), indicating that also in SGCs LAG3 expression might be a means to control an antitumor immune response. In conclusion, the results of our interdependency analysis show that LAG3 expression on TILs is upregulated in a setting of *TP53* mutations with different LAG3 expression patterns for nonsense and missense mutations.

Our multicolor stainings revealed a predominant co-expression of LAG3 and CD8 in TILs. Several studies demonstrated LAG3 expression on effector CD8+ TILs in different cancer types [7, 37]. Apparently, LAG3+ exerts immunosuppressive functions on CD8+ TILs even in the absence of CD4+ helper T cells [38]. Furthermore, we observed LAG3 expression on both CD4+ FOXP3+ and CD4+ FOXP3− T cells. While the former have been termed immunosuppressive Tregs, LAG3 expression on both immunophenotypes has been linked to inhibition of antitumor activity [5, 39]. We demonstrated intratumoral LAG3 expression in both the T helper and the effector T cell compartment. Studies concerning other tumor entities are in line with this finding and have revealed evidence for a functional impact of this expressional pattern. Thus, our data indicate that LAG3 plays an important role in the immunological TME of SGC.

In the univariate analysis, the expression of LAG3 did not significantly alter EFS for the overall cohort. Contrarily in the entity-wise univariate analysis, LAG3 positivity correlated with a decreased EFS in AdCy, while no prognostic effect was detected for the remaining histologic groups. This prognostic relevance is in line with previous studies investigating LAG3 in other tumor types [8, 40], even though studies with breast cancer came to divergent results [41, 42]. Using a multivariate cox analysis, we tested whether LAG3 expression might be a predictor of decreased EFS independent of the covariates age category, N- and T-stage, therapy, consumption of alcohol and nicotine. Indeed, we found a trend for shorter EFS in the LAG3-positive subgroup of AdCy. For the remaining variables, CD8 expression and TP53 mutation, we previously demonstrated a significant interdependency with LAG3 and consecutively excluded these variables from multivariate analysis. Hence, we cannot rule out that the prognostic effect of LAG3 expression could be partially attributed to these two cofactors.

Even though the impact of LAG3 expression on EFS was confined to the AdCy subgroup, we demonstrated that in the overall cohort, LAG3 expression significantly correlated with higher N-stage and UICC classification. This suggests that LAG3 might have a significant prognostic relevance also for other histotypes in a larger cohort.

Prognostic implications of different mutational patterns of TP53 expression are sparsely documented. While *TP53* mutations are widely regarded as predictors for a poor outcome, in most studies missense and nonsense mutations do not differ significantly regarding outcome [43]. We found only one study that reported a differential prognosis for nonsense compared to missense mutations: In this publication, nonsense mutations in SDC were associated with shorter progression-free survival than missense mutations [44]. Similarly, in our overall cohort, we demonstrated that *TP53* nonsense mutations are associated with a shorter EFS in univariate analyses (Kaplan–Meier model, univariate cox regression). Additionally, the multivariate cox regression analysis revealed a trend (*p* = 0.065) toward a shorter EFS for these mutations. Analogous to the above-mentioned procedure, here, we excluded the cofactor LAG3 for which we previously demonstrated a significant interdependency with TP53 mutations. Interestingly, we did not observe a comparable effect on the EFS for missense mutations, implicating profound differences of these mutation types for cancer aggressiveness. Comparable to our findings concerning LAG3 expression, TP53 mutations significantly correlated with higher T, N and UICC stage. This indicates that also *TP53* mutations might have a more pronounced prognostic relevance on EFS in a larger dataset.

Due to its retrospective and descriptive nature, our study cannot yield evidence for effectiveness of LAG3 inhibition in SGC. To investigate its therapeutic potential, also with respect to tumor mutational burden as a possible predictive marker, clinical studies are warranted. We did not examine the expression of other immunological markers such as PD-L1, CTLA-4 and TIM-3. Whether the co-expression of LAG3 with these molecules could have a more pronounced prognostic impact should be addressed by future studies. This could be of particular interest as synergistic LAG3/PD-L1 co-expression has been associated with T cell anergy [7]. Moreover, multiple trials evaluate the combinatory inhibition of LAG3 and other IRs [45].

In conclusion, the present study was intended to depict the entity-wise expression of LAG3 in a large cohort of SGC, a group of carcinomas bearing only sparse therapeutic targets. For the first time, we demonstrate that LAG3 is expressed on TILs in most SGC subtypes. In particular, LAG3 expression was frequently observed in aggressive tumor types, in carcinomas with lymphatic metastases and in *TP53*-mutated tumors, suggesting that LAG3 blockage could be of particular value for patients with an otherwise poor prognosis.

### Electronic supplementary material

Below is the link to the electronic supplementary material.

Supplementary material 1 (PDF 60 kb)

## Abbreviations

AdCy: Adenoid cystic carcinoma
ANOS: Adenocarcinoma not otherwise specified
CI: Confidence interval
CTLA-4: Cytotoxic T-lymphocyte-associated antigen 4
EFS: Event-free survival
FFPE: Formalin-fixed and paraffin-embedded
FISH: Fluorescence in situ hybridization
GOF: Gain of function
HNSCC: Head and neck squamous cell carcinoma
HR: Hazard ratio
IHC: Immunohistochemistry
IR: Inhibitory receptors
LAG3: Lymphocyte activation gene 3
MEC: Mucoepidermoid carcinoma
NSCLC: Nonsmall cell lung cancer
NT: Null type
OT: Overexpressed type
PD-1: Programmed cell death protein 1
PD-L1: Programmed death ligand 1
PD-L2: Programmed death ligand 2
SDC: Salivary duct carcinoma
SGC: Salivary gland carcinoma
TC: Tumor cells
TILs: Tumor-infiltrating lymphocytes
TMA: Tissue micro-array
TME: Tumor micro-environment
UICC: Union Internationale Contre le Cancer
WT: Wild type
