# Supplementary material for: Lymphocyte activation gene 3 (LAG3) protein expression on tumor-infiltrating lymphocytes in aggressive and TP53-mutated salivary gland carcinomas
Source: Cancer Immunol Immunother. 2020 Mar 30;69(7):1363–73. doi: 10.1007/s00262-020-02551-6 (PMC7370910; doi:10.1007/s00262-020-02551-6)
Supplement: Supplementary file 1 — Supplementary material 1 (PDF 60 kb) [file 262_2020_2551_MOESM1_ESM.pdf]

		LAG3 score				CD8 score			
		< 1% TILs	1-2% TILs	≥ 3% TILs	p	< 10 / spot	10-100 / spot	> 100 / spot	p
**TP53 mutation**	wildtype	55 (82.1)	6 (9)	6 (9)	**< 0.001** (n = 98)	18 (25.7)	37 (52.9)	15 (21.4)	n.s. (n = 101)
	nulltype	4 (33.3)	6 (50)	2 (16.7)		2 (16.7)	4 (33.3)	6 (50)	
	overexp.	11 (57.9)	1 (5.3)	7 (36.8)		3 (15.8)	11 (57.9)	5 (26.3)	
**CD8**	< 10 / spot	24 (92.3)	2 (7)	0 (0)	**< 0.001** (n = 103)				
	10-100 / spot	37 (74)	9 (18)	4 (8)					
	> 100 / spot	13 (48.1)	3 (11)	11 (40.7)					
**Total**		74 (71.8)	14 (13.6)	15 (14.6)		26 (24.5)	53 (50)	27 (25.5)	

Supplementary table 1: Interdependencies of LAG3, CD8 and *TP53* mutation types. LAG3 and CD8 expression displayed in three categories. Subgroup row percentages in parentheses.
